# *fac*-[*N*,*N*′-Bis(3-chloro-2-fluoro­benzyl­idene)ethyl­enediamine]bromido­tri­carbonyl­rhenium(I)

**DOI:** 10.1107/S1600536809001044

**Published:** 2009-01-14

**Authors:** Reza Kia, Hoong-Kun Fun

**Affiliations:** a X-ray Crystallography Unit, School of Physics, Universiti Sains Malaysia, 11800 USM, Penang, Malaysia

## Abstract

In the title compound, [ReBr(C_16_H_12_Cl_2_F_2_N_2_)(CO)_3_], the Re atom is in a slightly distorted octa­hedral coordination environment with the three carbonyl ligands having a *fac* configuration. The diimine ligand is equatorial and is bonded to the Re centre in an *N*,*N*′-bidentate chelating fashion, with a bite angle of 77.7 (2)°. The dihedral angle between the two benzene rings is 88.7 (6)°. In the crystal structure, there are F⋯O [2.856 (9) Å], Cl⋯C [3.150 (8) Å] and O⋯C [2.984 (10) Å] contacts which are shorter than the sum of the van der Waals radii for these atoms. In addition, symmetry-related mol­ecules are linked *via* inter­molecular C—H⋯O, C—H⋯Br and the F⋯O inter­actions into one-dimensional chains extending along the *a* axis. The crystal structure is further stabilized by inter­molecular π–π inter­actions [centroid–centroid distance = 3.571 (5) Å].

## Related literature

For values of standard bond lengths, see Allen *et al.* (1987 ▶). For related structures, see, for example: Kia *et al.* (2007 ▶). For backgroud to the applications of rhenium tricarbonyl diimine complexes, see, for example: Lee (1987 ▶); Farrell & Vlcek (2000 ▶); Collin & Sauvage (1989 ▶); Balzani *et al.* (1996 ▶).
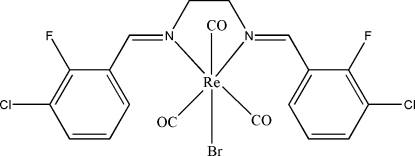


## Experimental

### 

#### Crystal data

[ReBr(C_16_H_12_Cl_2_F_2_N_2_)(CO)_3_]
*M* *_r_* = 691.32
Triclinic, 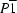

*a* = 7.3238 (3) Å
*b* = 12.3077 (4) Å
*c* = 13.1984 (5) Å
α = 116.504 (2)°
β = 99.707 (2)°
γ = 90.404 (2)°
*V* = 1044.84 (7) Å^3^
*Z* = 2
Mo *K*α radiation
μ = 8.03 mm^−1^
*T* = 100.0 (1) K
0.32 × 0.12 × 0.07 mm

#### Data collection

Bruker APEXII CCD area-detector diffractometer
Absorption correction: multi-scan (**SADABS**; Bruker, 2005 ▶) *T*_min_ = 0.170, *T*_max_ = 0.569
33300 measured reflections
9014 independent reflections
7668 reflections with *I* > 2σ(*I*)
*R*_int_ = 0.030

#### Refinement

*R*[*F* ^2^ > 2σ(*F* ^2^)] = 0.050
*wR*(*F* ^2^) = 0.154
*S* = 1.08
9014 reflections
265 parameters
H-atom parameters constrained
Δρ_max_ = 4.36 e Å^−3^
Δρ_min_ = −2.86 e Å^−3^

### 

Data collection: *APEX2* (Bruker, 2005 ▶); cell refinement: *SAINT* (Bruker, 2005 ▶); data reduction: *SAINT*; program(s) used to solve structure: *SHELXTL* (Sheldrick, 2008 ▶); program(s) used to refine structure: *SHELXTL*; molecular graphics: *SHELXTL*; software used to prepare material for publication: *SHELXTL* and *PLATON* (Spek, 2003 ▶).

## Supplementary Material

Crystal structure: contains datablocks global, I. DOI: 10.1107/S1600536809001044/lh2753sup1.cif

Structure factors: contains datablocks I. DOI: 10.1107/S1600536809001044/lh2753Isup2.hkl

Additional supplementary materials:  crystallographic information; 3D view; checkCIF report

## Figures and Tables

**Table:** 

Re1—C1	1.898 (7)
Re1—C3	1.911 (8)
Re1—C2	1.918 (7)
Re1—N2	2.190 (6)
Re1—N1	2.211 (6)
Re1—Br1	2.6564 (7)

**Table:** 

N2—Re1—N1	77.7 (2)

**Table 2:** Hydrogen-bond geometry (Å, °)

*D*—H⋯*A*	*D*—H	H⋯*A*	*D*⋯*A*	*D*—H⋯*A*
C8—H8*A*⋯Br1	0.93	2.80	3.691 (7)	161
C10—H10*A*⋯Br1^i^	0.93	2.93	3.845 (7)	170
C11—H11*B*⋯O3^i^	0.97	2.48	3.264 (10)	137

Symmetry code: (i) 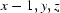.

**Fig. 1.**
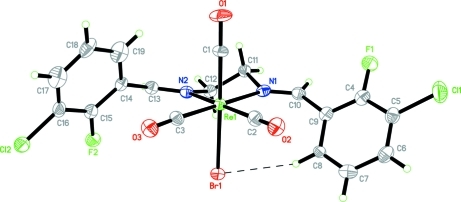
The molecular structure of (I), showing 50% probability displacement ellipsoids and the atomic numbering. Intramolecular interaction is shown as a dashed line.

**Fig. 2.**
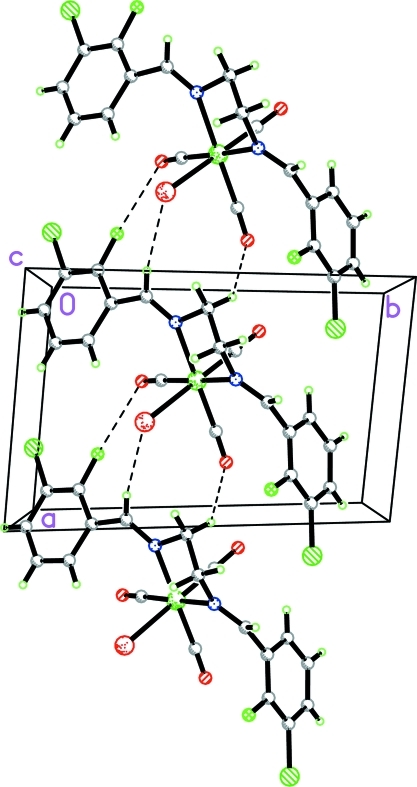
The crystal packing of (I), viewed along the c-axis showing a 1-D extended chain along the a-axis. Intermolecular interactions are shown as dashed lines.

## supplementary crystallographic information

### Comment

Rhenium tricarbonyl diimine complexes have been the subject of much attention, mainly because of their photophysical and photochemical properties (Lee 1987; Farrell & Vlcek 2000; Balzani et al. 1996) and their use in the photoreducetion and electroreduction of CO_2_ to CO (Collin & Sauvage 1989), a process of interest in the conversion and storage of solar energy. We report here the results of an X-ray structure determination of the title complex, (I).

In the title compound (I, Fig. 1), the Re atom is in a slightly distorted octahedral coordination environment. The bond lengths (Allen et al., 1987) and angles are within the normal ranges and are comparable to related structures (Kia et al., 2007). The three carbonyl ligands bonded to the Re atom are arranged in a fac configuration. The cis-equatorial bite angle [N1–Re1–N2] is 77.7 (2)°. The deviation of the Re atom from the mean plane defined by N1/N2/C2/C3 is 0.04 (4) Å. Due to the π-donor character of the bromine ligand, the length of the axial Re–C bond is slightly shorter than the values of the equatorial Re–C bonds (Table 1). In spite of the sp^2^ hybrizidation of the donor nitrogen atoms of the diimine ligand, the ReN_2_C_2_ five-membered chelate ring is significantly puckered which is reflected in the deviation from 120° for the Re1–N1–C10 and Re1–N2–C13 angles being 135.2 (5)° and 131.4 (6)°, respectively. Some interesting features of the crystal structure are the F1···O2 [2.860 (12) Å; symmetry code: -1 + x, y, z], O2···C18 [2.981 (14); symmetry code: 1 - x, 1 - y, -z] and Cl2···C13 [3.143 (12) Å; symmetry code: 1 + x, y, z] contacts which are shorter than the sum of the van der Waals radii of these atoms. In addition, symmetry-related molecules are linked via intermolecular C—H···O,  C—H···Br and C—F···O interactions into 1-D extended chains along the a-axis (Table 2, Fig. 2). The crystal structure is further stabilized by intramolecular C—H···Br and intermolecular π-π interactions [Cg1···Cg1 = 3.571 (5) Å; symmetry code: -x, -y, -z; Cg1 is the centroid of the C4–C9 benzene ring].

### Experimental

The synthetic method has been described earlier (Kia *et al.,* 2007), except that *N,N'*-bis(3-chloro-2-fluoro-benzylidene) ethylenediamine ligand and [Re(CO)_5_Br] were used as starting materials. Single crystals suitable for *X*-ray diffraction were obtained by evaporation of an dichloromethane/toluene (4/1 ratio) solution at room temperature.

### Refinement

All hydrogen atoms were positioned geometrically and refined in a riding approximation model with C–H = 0.93-0.97 Å and U_iso_ (H) = 1.2 U_eq_ (C). The highest peak (4.36 eÅ^-3^) is located 1.76 Å from Cl1 and the deepest hole (-2.86 eÅ^-3^) is located 1.17 Å from C15.

### Crystal data

**Table:**

[ReBr(C_16_H_12_Cl_2_F_2_N_2_)(CO)_3_]	*Z* = 2
*M**_r_* = 691.32	*F*(000) = 652
Triclinic, *P*1	*D*_x_ = 2.197 Mg m^−^^3^
Hall symbol: -P 1	Mo *K*α radiation, λ = 0.71073 Å
*a* = 7.3238 (3) Å	Cell parameters from 9990 reflections
*b* = 12.3077 (4) Å	θ = 3.1–36.5°
*c* = 13.1984 (5) Å	µ = 8.03 mm^−^^1^
α = 116.504 (2)°	*T* = 100 K
β = 99.707 (2)°	Block, yellow
γ = 90.404 (2)°	0.32 × 0.12 × 0.07 mm
*V* = 1044.84 (7) Å^3^	

### Data collection

**Table:**

Bruker SMART APEXII CCD area-detector diffractometer	9014 independent reflections
Radiation source: fine-focus sealed tube	7668 reflections with *I* > 2σ(*I*)
graphite	*R*_int_ = 0.030
φ and ω scans	θ_max_ = 35.0°, θ_min_ = 2.8°
Absorption correction: multi-scan (*SADABS*; Bruker, 2005)	*h* = −11→10
*T*_min_ = 0.170, *T*_max_ = 0.569	*k* = −19→19
33300 measured reflections	*l* = −21→21

### Refinement

**Table:**

Refinement on *F*^2^	Primary atom site location: structure-invariant direct methods
Least-squares matrix: full	Secondary atom site location: difference Fourier map
*R*[*F*^2^ > 2σ(*F*^2^)] = 0.050	Hydrogen site location: inferred from neighbouring sites
*wR*(*F*^2^) = 0.154	H-atom parameters constrained
*S* = 1.08	*w* = 1/[σ^2^(*F*_o_^2^) + (0.043*P*)^2^ + 25.3509*P*] where *P* = (*F*_o_^2^ + 2*F*_c_^2^)/3
9014 reflections	(Δ/σ)_max_ = 0.001
265 parameters	Δρ_max_ = 4.36 e Å^−^^3^
0 restraints	Δρ_min_ = −2.86 e Å^−^^3^

### Special details

**Table:**

Experimental. The low-temperature data was collected with the Oxford Cyrosystem Cobra low-temperature attachment.
Geometry. All esds (except the esd in the dihedral angle between two l.s. planes) are estimated using the full covariance matrix. The cell esds are taken into account individually in the estimation of esds in distances, angles and torsion angles; correlations between esds in cell parameters are only used when they are defined by crystal symmetry. An approximate (isotropic) treatment of cell esds is used for estimating esds involving l.s. planes.
Refinement. Refinement of F^2^ against ALL reflections. The weighted R-factor wR and goodness of fit S are based on F^2^, conventional R-factors R are based on F, with F set to zero for negative F^2^. The threshold expression of F^2^ > 2sigma(F^2^) is used only for calculating R-factors(gt) etc. and is not relevant to the choice of reflections for refinement. R-factors based on F^2^ are statistically about twice as large as those based on F, and R- factors based on ALL data will be even larger.

### Fractional atomic coordinates and isotropic or equivalent isotropic displacement parameters (Å^2^)

**Table:**

	*x*	*y*	*z*	*U*_iso_*/*U*_eq_	
Re1	0.41651 (4)	0.44687 (2)	0.17913 (2)	0.01814 (7)	
Br1	0.59914 (10)	0.30823 (6)	0.26094 (6)	0.02132 (13)	
Cl1	−0.2702 (4)	−0.0324 (3)	−0.2767 (2)	0.0453 (6)	
Cl2	1.1905 (3)	0.8265 (2)	0.5091 (2)	0.0379 (5)	
F1	−0.2477 (7)	0.1706 (5)	−0.0492 (5)	0.0332 (10)	
F2	0.8904 (7)	0.6865 (5)	0.5305 (5)	0.0304 (10)	
O1	0.1897 (9)	0.6185 (6)	0.1033 (7)	0.0365 (14)	
O2	0.4182 (9)	0.2812 (6)	−0.0766 (5)	0.0291 (11)	
O3	0.7869 (8)	0.5564 (6)	0.1675 (5)	0.0293 (11)	
N1	0.1581 (9)	0.3695 (6)	0.1978 (5)	0.0216 (10)	
N2	0.4113 (9)	0.5578 (6)	0.3623 (5)	0.0215 (10)	
C1	0.2797 (10)	0.5521 (7)	0.1317 (6)	0.0236 (12)	
C2	0.4139 (10)	0.3398 (7)	0.0196 (6)	0.0240 (12)	
C3	0.6462 (10)	0.5171 (7)	0.1739 (6)	0.0231 (12)	
C4	−0.0837 (11)	0.1231 (7)	−0.0633 (7)	0.0260 (13)	
C5	−0.0743 (12)	0.0240 (7)	−0.1673 (7)	0.0275 (14)	
C6	0.0927 (13)	−0.0300 (7)	−0.1816 (7)	0.0293 (15)	
H6A	0.1011	−0.0974	−0.2506	0.035*	
C7	0.2454 (12)	0.0175 (8)	−0.0925 (7)	0.0304 (15)	
H7A	0.3565	−0.0185	−0.1019	0.036*	
C8	0.2359 (10)	0.1165 (6)	0.0091 (6)	0.0209 (11)	
H8A	0.3399	0.1465	0.0683	0.025*	
C9	0.0687 (10)	0.1738 (7)	0.0249 (6)	0.0230 (12)	
C10	0.0427 (10)	0.2764 (7)	0.1340 (6)	0.0237 (12)	
H10A	−0.0681	0.2728	0.1584	0.028*	
C11	0.1005 (10)	0.4586 (6)	0.3060 (6)	0.0219 (12)	
H11A	0.0068	0.4196	0.3259	0.026*	
H11B	0.0491	0.5263	0.2959	0.026*	
C12	0.2731 (10)	0.5034 (7)	0.4001 (6)	0.0222 (12)	
H12A	0.2437	0.5638	0.4720	0.027*	
H12B	0.3218	0.4361	0.4121	0.027*	
C13	0.5117 (10)	0.6545 (7)	0.4396 (6)	0.0231 (12)	
H13A	0.5006	0.6803	0.5159	0.028*	
C14	0.6436 (10)	0.7276 (6)	0.4158 (6)	0.0215 (11)	
C15	0.8330 (10)	0.7421 (7)	0.4645 (6)	0.0237 (12)	
C16	0.9615 (11)	0.8108 (7)	0.4457 (7)	0.0270 (14)	
C17	0.8965 (15)	0.8671 (9)	0.3764 (7)	0.0369 (13)	
H17A	0.9809	0.9116	0.3607	0.044*	
C18	0.7106 (15)	0.8577 (8)	0.3315 (7)	0.0354 (19)	
H18A	0.6697	0.9000	0.2898	0.042*	
C19	0.5845 (16)	0.7865 (9)	0.3474 (7)	0.0369 (13)	
H19A	0.4598	0.7770	0.3130	0.044*	

### Atomic displacement parameters (Å^2^)

**Table:**

	*U*^11^	*U*^22^	*U*^33^	*U*^12^	*U*^13^	*U*^23^
Re1	0.01889 (11)	0.01916 (11)	0.01800 (11)	0.00236 (8)	0.00407 (8)	0.00967 (8)
Br1	0.0215 (3)	0.0235 (3)	0.0214 (3)	0.0055 (2)	0.0059 (2)	0.0116 (2)
Cl1	0.0397 (12)	0.0435 (12)	0.0334 (10)	−0.0032 (9)	−0.0028 (9)	0.0041 (9)
Cl2	0.0277 (9)	0.0276 (9)	0.0590 (14)	0.0045 (7)	0.0142 (9)	0.0180 (9)
F1	0.025 (2)	0.032 (2)	0.039 (3)	0.0052 (19)	0.0086 (19)	0.012 (2)
F2	0.029 (2)	0.029 (2)	0.040 (3)	0.0031 (18)	0.0048 (19)	0.022 (2)
O1	0.033 (3)	0.040 (3)	0.053 (4)	0.015 (3)	0.012 (3)	0.035 (3)
O2	0.035 (3)	0.028 (3)	0.023 (2)	0.002 (2)	0.008 (2)	0.010 (2)
O3	0.027 (3)	0.035 (3)	0.031 (3)	0.001 (2)	0.006 (2)	0.019 (2)
N1	0.024 (3)	0.021 (2)	0.023 (3)	0.006 (2)	0.008 (2)	0.010 (2)
N2	0.022 (3)	0.022 (3)	0.023 (2)	0.005 (2)	0.007 (2)	0.011 (2)
C1	0.023 (3)	0.025 (3)	0.027 (3)	0.006 (2)	0.004 (2)	0.016 (3)
C2	0.023 (3)	0.026 (3)	0.024 (3)	0.002 (2)	0.006 (2)	0.012 (3)
C3	0.023 (3)	0.026 (3)	0.020 (3)	0.002 (2)	0.003 (2)	0.011 (2)
C4	0.027 (3)	0.025 (3)	0.028 (3)	0.000 (3)	0.009 (3)	0.013 (3)
C5	0.031 (4)	0.024 (3)	0.026 (3)	0.000 (3)	0.006 (3)	0.011 (3)
C6	0.040 (4)	0.021 (3)	0.028 (3)	0.002 (3)	0.011 (3)	0.010 (3)
C7	0.031 (4)	0.034 (4)	0.032 (4)	0.012 (3)	0.012 (3)	0.019 (3)
C8	0.020 (3)	0.017 (3)	0.026 (3)	0.001 (2)	0.003 (2)	0.011 (2)
C9	0.024 (3)	0.021 (3)	0.024 (3)	0.000 (2)	0.008 (2)	0.009 (2)
C10	0.021 (3)	0.024 (3)	0.027 (3)	0.004 (2)	0.008 (2)	0.011 (3)
C11	0.022 (3)	0.021 (3)	0.023 (3)	0.005 (2)	0.007 (2)	0.009 (2)
C12	0.023 (3)	0.024 (3)	0.024 (3)	0.004 (2)	0.009 (2)	0.013 (2)
C13	0.024 (3)	0.024 (3)	0.022 (3)	0.004 (2)	0.006 (2)	0.010 (2)
C14	0.026 (3)	0.018 (3)	0.020 (3)	0.000 (2)	0.005 (2)	0.008 (2)
C15	0.023 (3)	0.022 (3)	0.025 (3)	0.002 (2)	0.006 (2)	0.010 (2)
C16	0.027 (3)	0.019 (3)	0.030 (3)	0.000 (2)	0.009 (3)	0.005 (3)
C17	0.052 (4)	0.032 (3)	0.023 (2)	−0.001 (3)	0.010 (2)	0.009 (2)
C18	0.060 (6)	0.023 (3)	0.024 (3)	−0.003 (3)	0.004 (3)	0.012 (3)
C19	0.052 (4)	0.032 (3)	0.023 (2)	−0.001 (3)	0.010 (2)	0.009 (2)

### Geometric parameters (Å, °)

**Table:**

Re1—C1	1.898 (7)	C7—C8	1.364 (11)
Re1—C3	1.911 (8)	C7—H7A	0.9300
Re1—C2	1.918 (7)	C8—C9	1.417 (10)
Re1—N2	2.190 (6)	C8—H8A	0.9300
Re1—N1	2.211 (6)	C9—C10	1.476 (10)
Re1—Br1	2.6564 (7)	C10—H10A	0.9300
Cl1—C5	1.737 (9)	C11—C12	1.514 (10)
Cl2—C16	1.711 (9)	C11—H11A	0.9700
F1—C4	1.344 (9)	C11—H11B	0.9700
F2—C15	1.347 (9)	C12—H12A	0.9700
O1—C1	1.201 (9)	C12—H12B	0.9700
O2—C2	1.153 (9)	C13—C14	1.478 (10)
O3—C3	1.167 (9)	C13—H13A	0.9300
N1—C10	1.273 (10)	C14—C15	1.401 (10)
N1—C11	1.494 (9)	C14—C19	1.408 (12)
N2—C13	1.284 (10)	C15—C16	1.385 (11)
N2—C12	1.476 (9)	C16—C17	1.402 (13)
C4—C9	1.376 (11)	C17—C18	1.372 (15)
C4—C5	1.384 (11)	C17—H17A	0.9300
C5—C6	1.397 (12)	C18—C19	1.373 (13)
C6—C7	1.382 (13)	C18—H18A	0.9300
C6—H6A	0.9300	C19—H19A	0.9300
			
C1—Re1—C3	91.3 (3)	C4—C9—C8	117.8 (7)
C1—Re1—C2	88.3 (3)	C4—C9—C10	117.9 (7)
C3—Re1—C2	84.4 (3)	C8—C9—C10	124.0 (7)
C1—Re1—N2	94.3 (3)	N1—C10—C9	126.0 (7)
C3—Re1—N2	98.8 (3)	N1—C10—H10A	117.0
C2—Re1—N2	175.8 (3)	C9—C10—H10A	117.0
C1—Re1—N1	90.7 (3)	N1—C11—C12	107.0 (6)
C3—Re1—N1	176.1 (3)	N1—C11—H11A	110.3
C2—Re1—N1	99.0 (3)	C12—C11—H11A	110.3
N2—Re1—N1	77.7 (2)	N1—C11—H11B	110.3
C1—Re1—Br1	175.6 (2)	C12—C11—H11B	110.3
C3—Re1—Br1	90.5 (2)	H11A—C11—H11B	108.6
C2—Re1—Br1	95.9 (2)	N2—C12—C11	107.3 (6)
N2—Re1—Br1	81.48 (16)	N2—C12—H12A	110.3
N1—Re1—Br1	87.24 (16)	C11—C12—H12A	110.3
C10—N1—C11	115.3 (6)	N2—C12—H12B	110.3
C10—N1—Re1	135.2 (5)	C11—C12—H12B	110.3
C11—N1—Re1	109.0 (4)	H12A—C12—H12B	108.5
C13—N2—C12	117.3 (6)	N2—C13—C14	124.7 (7)
C13—N2—Re1	131.3 (5)	N2—C13—H13A	117.7
C12—N2—Re1	111.3 (4)	C14—C13—H13A	117.7
O1—C1—Re1	178.3 (7)	C15—C14—C19	118.4 (7)
O2—C2—Re1	175.8 (7)	C15—C14—C13	119.6 (6)
O3—C3—Re1	177.7 (7)	C19—C14—C13	122.0 (7)
F1—C4—C9	119.6 (7)	F2—C15—C16	119.6 (7)
F1—C4—C5	118.3 (7)	F2—C15—C14	118.7 (6)
C9—C4—C5	122.1 (8)	C16—C15—C14	121.7 (7)
C4—C5—C6	119.1 (8)	C15—C16—C17	118.0 (8)
C4—C5—Cl1	119.7 (7)	C15—C16—Cl2	119.4 (7)
C6—C5—Cl1	121.2 (6)	C17—C16—Cl2	122.6 (7)
C7—C6—C5	119.4 (7)	C18—C17—C16	121.2 (9)
C7—C6—H6A	120.3	C18—C17—H17A	119.4
C5—C6—H6A	120.3	C16—C17—H17A	119.4
C8—C7—C6	121.2 (8)	C17—C18—C19	120.6 (9)
C8—C7—H7A	119.4	C17—C18—H18A	119.7
C6—C7—H7A	119.4	C19—C18—H18A	119.7
C7—C8—C9	120.4 (7)	C18—C19—C14	120.1 (10)
C7—C8—H8A	119.8	C18—C19—H19A	120.0
C9—C8—H8A	119.8	C14—C19—H19A	120.0
			
C1—Re1—N1—C10	95.2 (8)	C7—C8—C9—C10	176.2 (7)
C2—Re1—N1—C10	6.9 (8)	C11—N1—C10—C9	−178.1 (7)
N2—Re1—N1—C10	−170.6 (8)	Re1—N1—C10—C9	11.1 (12)
Br1—Re1—N1—C10	−88.7 (7)	C4—C9—C10—N1	−142.2 (8)
C1—Re1—N1—C11	−76.0 (5)	C8—C9—C10—N1	44.3 (12)
C2—Re1—N1—C11	−164.3 (5)	C10—N1—C11—C12	140.9 (7)
N2—Re1—N1—C11	18.2 (4)	Re1—N1—C11—C12	−46.0 (6)
Br1—Re1—N1—C11	100.1 (4)	C13—N2—C12—C11	141.5 (7)
C1—Re1—N2—C13	−81.1 (7)	Re1—N2—C12—C11	−41.9 (6)
C3—Re1—N2—C13	10.9 (7)	N1—C11—C12—N2	58.1 (7)
N1—Re1—N2—C13	−170.9 (7)	C12—N2—C13—C14	−173.8 (6)
Br1—Re1—N2—C13	100.1 (7)	Re1—N2—C13—C14	10.4 (11)
C1—Re1—N2—C12	102.9 (5)	N2—C13—C14—C15	−119.2 (8)
C3—Re1—N2—C12	−165.1 (5)	N2—C13—C14—C19	62.3 (11)
N1—Re1—N2—C12	13.2 (4)	C19—C14—C15—F2	179.7 (7)
Br1—Re1—N2—C12	−75.9 (4)	C13—C14—C15—F2	1.2 (10)
F1—C4—C5—C6	−177.1 (7)	C19—C14—C15—C16	−0.9 (11)
C9—C4—C5—C6	3.0 (12)	C13—C14—C15—C16	−179.4 (7)
F1—C4—C5—Cl1	2.0 (10)	F2—C15—C16—C17	179.9 (7)
C9—C4—C5—Cl1	−177.9 (6)	C14—C15—C16—C17	0.5 (11)
C4—C5—C6—C7	−0.9 (12)	F2—C15—C16—Cl2	−1.4 (10)
Cl1—C5—C6—C7	−180.0 (7)	C14—C15—C16—Cl2	179.2 (6)
C5—C6—C7—C8	−0.2 (13)	C15—C16—C17—C18	2.0 (12)
C6—C7—C8—C9	−0.8 (12)	Cl2—C16—C17—C18	−176.7 (7)
F1—C4—C9—C8	176.3 (7)	C16—C17—C18—C19	−4.0 (14)
C5—C4—C9—C8	−3.9 (11)	C17—C18—C19—C14	3.6 (13)
F1—C4—C9—C10	2.4 (11)	C15—C14—C19—C18	−1.2 (12)
C5—C4—C9—C10	−177.8 (7)	C13—C14—C19—C18	177.3 (8)
C7—C8—C9—C4	2.7 (11)		

### Hydrogen-bond geometry (Å, °)

**Table:**

*D*—H···*A*	*D*—H	H···*A*	*D*···*A*	*D*—H···*A*
C8—H8A···Br1	0.93	2.80	3.691 (7)	161
C10—H10A···Br1^i^	0.93	2.93	3.845 (7)	170
C11—H11B···O3^i^	0.97	2.48	3.264 (10)	137

Symmetry codes: (i) *x*−1, *y*, *z*.
